# Minimal Residual Disease in Acute Myeloid Leukemia of Adults: Determination, Prognostic Impact and Clinical Applications

**DOI:** 10.4084/MJHID.2016.052

**Published:** 2016-10-20

**Authors:** Maria Ilaria Del Principe, Francesco Buccisano, Luca Maurillo, Giuseppe Sconocchia, Mariagiovanna Cefalo, Maria Irno Consalvo, Chiara Sarlo, Consuelo Conti, Giovanna De Santis, Eleonora De Bellis, Ambra Di Veroli, Patrizia Palomba, Cristina Attrotto, Annagiulia Zizzari, Giovangiacinto Paterno, Maria Teresa Voso, Giovanni Del Poeta, Francesco Lo-Coco, William Arcese, Sergio Amadori, Adriano Venditti

**Affiliations:** 1 Ematologia, Dipartimento di Biomedicina e Prevenzione, Università degli studi di Roma “Tor Vergata”, Roma, Italia; 2 Istituto di Farmacologia Translazionale, Dipartimento di Medicina, CNR, Roma, Italia; 3 Ematologia, Policlinico Universitario-Campus Biomedico, Roma, Italia; 4 Laboratorio di Neuro-Oncoematologia, I.R.C.C.S.- Fondazione S. Lucia, Roma, Italia

## Abstract

Pretreatment assessment of cytogenetic/genetic signature of acute myeloid leukemia (AML) has been consistently shown to play a major prognostic role but also to fail at predicting outcome on individual basis, even in low-risk AML. Therefore, we are in need of further accurate methods to refine the patients’ risk allocation process, distinguishing more adequately those who are likely to recur from those who are not. In this view, there is now evidence that the submicroscopic amounts of leukemic cells (called minimal residual disease, MRD), measured during the course of treatment, indicate the quality of response to therapy. Therefore, MRD might serve as an independent, additional biomarker to help to identify patients at higher risk of relapse. Detection of MRD requires the use of highly sensitive ancillary techniques, such as polymerase chain reaction (PCR) and multiparametric flow cytometry(MPFC). In the present manuscript, we will review the current approaches to investigate MRD and its clinical applications in AML management.

## Introduction

Acute myeloid leukemia (AML) is a clonal disorder of haemopoietic stem cells characterized by an abnormal proliferation of myeloid progenitors and subsequent bone marrow failure. AML response to chemotherapy is extremely variable with complete remission (CR) rates ranging from 50% to 80%.^1^ Also frequency of relapse is variable, being reported in 10% to 95% of the cases.^2^–^4^ Currently, risk-stratification is determined by several factors, patient- and disease-related, assessed at diagnosis, such as age, performance status, white blood count (WBC), existence of prior myelodysplastic syndrome, previous cytotoxic therapy for another disorder and cytogenetic/molecular abnormalities. Among long-established prognostic factors, karyotype and genotype of leukemic cells are the strongest for they impact on response to induction therapy and survival.^5^ However, cytogenetic and molecular findings at diagnosis allow stratification of ~40% of patients in “good risk” or “adverse risk” groups. The lack of cytogenetic and molecular markers in approximately 60% of AML, prompts the need for further accurate methods to select more precisely patients at high risk of disease recurrence. In this view, there is now evidence that levels of minimal residual disease (MRD) during the course of therapy could serve as an independent biomarker to identify such high-risk patients^6^,^7^ and to plan the therapeutic program accordingly. We will review the current approaches to investigate MRD and its clinical applications in AML management.

## MRD Detection

The paradigm of a successful treatment of AML is based on the achievement of morphological CR (mCR), defined as less than 5% leukemic cells, counted by light microscopy, within a fully restored bone marrow cellularity. However, it is now clear that classical morphology examination neglects a minority of myeloid blasts that could survive after induction and consolidation cycles. In this view, sophisticated techniques such as polymerase chain reaction (PCR) and multiparametric flow cytometry(MPFC) have been shown to detect leukemic cells at high sensitivity, in conditions of mCR. It is still a matter of debate what is the best method, in terms of clinical usefulness, to measure MRD in AML.

## MRD Detection by PCR

In general, PCR is regarded as the most sensitive technique with a detection power of 10^−4^ to10^−6^.^7^-^10^ Using PCR, MRD can be monitored by capturing specific leukemia targets such as chimeric fusion genes, mutations and gene overexpression (Table 1).

### Leukemic Fusion Genes

This approach relies on cloning of breakpoints of the chromosomal rearrangements in AML by using reverse transcriptase PCR (RT-PCR) or quantitative real-time PCR (RQ-PCR). In the situation of mCR, it allows identification of residual fusion genes in approximately 30% of the patients. Common targets for PCR-based MRD detection are fusion transcripts of mixed-lineage leukemia (MLL)-gene and CBF positive AML, e.g. runt-related transcription factor 1/runt–related transcription factor 1 translocated to 1 (RUNX1-RUNX1T1, formerly AML1-ETO) and core-binding factor subunit beta-myosin heavy chain 11 (CBFB-MYH11). Scholl et al.^11^,^12^ showed that patients achieving a MLL-AF9 PCR-negative state had a very low probability of relapse and a 4-year overall survival (OS) of 70%, whereas all of those with an RT-PCR positive finding relapsed and died within 3 years. UK MRC trials group demonstrated that in CBF positive AML, MRD monitoring by RT-PCR at different time points identified patients at higher risk of relapse.^9^ However, using RT-PCR, persistent PCR positivity has been observed in long-term survivors even after allogeneic stem cell transplantation (ASCT). Therefore, RT-PCR in CBF positive AML may have a limited clinical applicability since the detection of low transcript levels in a situation of long-term remission is not likely to anticipate an impending relapse. Indeed, long-term persistence of CBF RT-PCR signal would reflect the successful immune surveillance or the presence of MRD target in the leukemic stem cells (LCS) that requires additional genetic hits for progression to overt disease.^13^ In this view, RQ-PCR is potentially more advantageous than RT-PCR owing to its capability to predict impending relapse during long-term follow-up monitoring.^14^ Corbacioglu et al., 15 using RQ-PCR, established clinically relevant MRD checkpoints in which persistence of CBFB-MYH11 transcript positivity singled out patients with significantly increased the risk of relapse. The authors concluded that monitoring of CBFB-MYH11 transcript levels should be incorporated into future clinical trials to guide therapeutic decisions. In a prospective multicenter trial, Jourdan et al.^16^ demonstrated, by RQ-PCR, that a less than 3-log MRD reduction of RUNX1-RUNX1T1 transcript after the first consolidation was associated with a higher specific hazard of relapse in young CBF-AML patients. At 36 months, the cumulative incidence of relapse (CIR) and relapse-free survival (RFS) was lower and longer, respectively, in patients who achieved 3-log MRD reduction. A decline of RUNX1-RUNX1T1 transcript inferior to 3 logs after 2 courses of consolidation or within 3–4 months after mCR, were found to predict relapse in other studies.^17^,^18^ A further multicenter prospective cohort study confirmed the threshold of >3-log reduction and indicated the second consolidation as the best timing for MRD examination.^19^

### Mutations

Fusion genes are present in about 30% of AML cases. In fusion gene negative AML patients, possible targets for PCR-based MRD assessment are Fms-like tyrosine kinase- internal tandem duplication (FLT3-ITD), mutated nucleophosmin1 (NPM1),and DNA methyltransferase(DNMT3A). About 25% of AML patients carried FLT3-ITD that predicts poor outcome especially when it is located in the tyrosine kinase domain.^20^ Several tyrosine kinase inhibitors are currently under investigation since FLT3 could be a meaningful, actionable therapeutic target AML.^21^ In light of this, detection of MRD by monitoring this marker would be useful to measure the anti-leukemic activity of FLT3 inhibitors. However, mutational shifts between diagnosis and relapse, multiclonality at presentation, the outgrowth of clones at relapse different from those detected at diagnosis, variable insertion sites, and lengths among patients, make the use of FLT3 mutation still unreliable for MRD monitoring.^22^,^23^ There is evidence that the lack of longitudinal stability of gene mutations reflects the insufficient sensitivity of the currently used methodologies. Next-generation sequencing (NGS), with its increased sensitivity, might pave the way to a more accurate MRD monitoring of FLT3-ITD in AML patients.^24^–^26^ In this regard, Zuffa et al.^27^ developed an amplicon based-ultra deep sequencing (UDS ) approach for FLT3 mutational screening that revealed the presence of small ITD+ clones in 5 of 256 normal karyotypes (CN-) AML patients, who were *FLT3* wild-type at presentation, but tested ITD+ at relapse or disease progression. Thus, UDS appears as a valuable tool not only for FLT3 mutational screening but also MRD monitoring. NPM1 mutations are very stable at relapse^28^ thus that they might have a role in MRD assessment. NPM1 gene mutations are present in 30% of all AML patients and in 50% of those with CN.^29^ Several studies have shown a favorable impact of NPM1-mutated (NPM1^mut^) on clinical outcome in the CN-AML setting.^20^,^29^ Nevertheless, a substantial proportion of patients with NPM1 mutations will eventually experience a disease recurrence. In a retrospective analysis performed on 155 patients, increasing MRD levels of NPM1 were predictive of relapse after chemotherapy or allogeneic hematopoietic stem cell transplantation (ASCT).^30^ These data are in concordance with previous reports investigating comparable data sets. Schnittger et al.^31^ developed a highly sensitive RQ-PCR assay able to prime 17 different mutations of NPM1. In 252 NPM1^mut^AML, high levels of NPM1^mut^ were significantly correlated with outcome, at each of four time-points of monitoring. In multivariate analysis, including age, FLT3-ITD status and the level of residual NPM1, it was demonstrated that the latter was the most relevant prognostic factor affecting event free survival (EFS) during first-line treatment, also in the subgroup of patients undergoing ASCT. In a further refinement of such an approach, Kronke et al.^32^ demonstrated that NPM1^mut^transcripts levels measured at two distinct checkpoints, after double induction and consolidation therapy, impacted on OS and CIR (p<0.001 for all comparisons). Recently, Ivey et al.^33^ confirmed the prognostic role of residual NPM1^mut^ transcripts. After the second cycle of chemotherapy, the persistence of NPM1^mut^ transcripts was observed in the peripheral blood of 15% previously untreated patients. Such a persistence was associated with a 3-year greater risk of relapse (82% vs. 30%) and a lower rate of survival (24% vs. 75%) than in a situation of transcript undetectability. In multivariate analysis, the presence in the peripheral blood of MRD was the only independent prognostic factor associated with death. Another possible target for MRD monitoring is DNMT3A, found in 15–25% of AML patients, particularly in CN AML patients.^34^,^35^ The presence of DNMT3A mutations is an independent determinant of dismal prognosis both in the overall population and high-risk category (FLT3-ITD, age older than 60 years).^34^ To explore the utility of DNMT3A mutations as biomarkers for MRD in AML, Pløen et al.^36^ developed assays for sensitive detection of recurrent mutations affecting residue R882. Analysis of DNA from 298 diagnostic AML samples revealed DNMT3A mutations in 45 cases (15%), which coincided with mutations in NPM1, FLT3 and isocitrate dehydrogenase 1. DNMT3A mutations were stable in 12 of 13 patients presenting with relapse or secondary myelodysplastic syndrome, but were also present in remission samples of 14 patients until 8 years after initial AML diagnosis, despite the loss of all other molecular AML markers. Based on these data, the suitability of DNMT3A as MRD marker is still questioned.

### Gene overexpression

MRD can also be monitored through detection of gene overexpression. Several genes have been proposed as candidates, with Wilm’s Tumor gene (WT1) being the most reliable. WT1 is a tumor suppressor gene that encodes for a zinc-finger transcription factor that is aberrantly overexpressed in 85–90% of AML cases.^10^ The value of WT1 monitoring in AML has been a matter of debate, mainly due to differences among the assays in use. This led to the development of a standardized WT1 assay, validation of which involved a network of 11 laboratories and provided independent prognostic information in AML. Among a cohort of 129 AML patients, a WT1 reduction below 200 copies after the first induction chemotherapy was associated with a longer duration of CR, independently from age, WBC count or cytogenetic risk group.^10^ Based on the post induction WT1 level, Nomdedeu et al.^37^ identified three prognostic AML groups: group 0 (no. ofWT1 copies 0–17.5, in 134 patients), group 1 (no. ofWT1 copies 17.6–170.5, in 160 patients), and group 3 (no. of WT1 copies >170.5, in 71 patients). Outcomes of these groups differed significantly in terms of OS (59±4%, 59±4%, 72±5%), leukemia free survival (24±7%, 46±4%, 65±5%) and relapse probability (CIR 72±4%, 45±4%,25±5%). In line with these data, the RQ-PCR positivity of WT1-MRD (defined as >0.5% in peripheral blood) after induction, was associated with a higher risk of relapse and a shorter OS in a further series of 183 AML patients with WT1 overexpression.^38^ The post induction time-point was confirmed in 45 AML patients, in whom a post-inductionWT1 log clearance < 1.96 predicted disease recurrence.^39^ Levels of WT1 higher than 150 copies/10^4^ABL after induction course are associated with a shorter RFS, also in childhood AML patients.^40^ Furthermore, Pozzi et al. found that WT1 expression>100 copies predicted relapse even after ASCT. Actually, patients who received donor lymphocyte infusion after ASCT, because of high WT1 levels, had an OS significantly longer than those who expressed the same high levels but were not given donor lymphocytes.^41^ Finally, there is evidence that the presence of high levels of WT1 gene in circulating RNA after ASCT predicts AML recurrence.^42^ Moreover, WT1 was listed as the theoretically best single universal molecular marker for MRD detection in AML.^10^,^42^ In practice its monitoring cannot be applied in all AML cases, which can exhibit significantly different patterns of expression.^43^,^44^ Furthermore, since the expression of WT1 is not leukemia-specific, discriminating genuine residual disease from background expression can be problematic.^6^,^38^ In order to mitigate the limitations of this promising but sub-optimally used marker for MRD detection, Goswami et al.^45^ developed a technique based on the identification of a panel of genes, including WT1, which are overexpressed in AML. They concluded that multiple gene based MRD assay was superior to the use of WT1 alone for MRD purposes. In fact, this approach allowed WT1 MRD negative patients to be reclassified as positive on the basis of the measure of other genes.

## MRD Detection by MPFC

MPFC provides a quick and relatively inexpensive method for MRD detection, which is applicable to the vast majority of patients with AML. In fact,≥ 85% of AML cases exhibitsan aberrant phenotype called “leukemia-associated immunophenotype” (LAIP). LAIP is defined as the combination of antigens and/or flow-cytometry physical abnormalities that are absent or very infrequent in healthy bone marrow.^46^ Phenotypic abnormalities in AML include expression of markers not expressed on myeloid cells (lymphoid-affiliated antigens such as CD7, CD19, and CD56), co-expression of markers commonly expressed at different stages of maturation as well as over-expression and under-expression of myeloid markers (e.g. CD33)(Table 2).^47^,^48^ Initial studies of normal and leukemic phenotypes were performed in 2–3 color-assays.^47^,^49^ With the time it became manifest that implementing LAIP identification required a more comprehensive diagnostic antibody panel. In this regard, international efforts are being made to generate standardized MPFC protocols, which cover the phenotypic heterogeneity of AML and the large number of potential LAIPs.^50^,^51^ Actually, the diffusion of devices equipped with multiple lasers has implemented multiple color assays (>6–10 monoclonal antibody combinations) thus favoring increment of sensitivity from 10^−3^ to 10^−5^.^52^-^54^ Accordingly, MPFC appears a highly sensitive and specific method to monitor MRD in AML patients. Transposition of MPFC approach to the clinical reality, requires that key-issues, such as MRD thresholds and appropriate time-points to determine MRD, are adequately addressed. Ideally, threshold and time-point should be the ones, assessment of which provides the most informative prognostic indication, thus that the choice of post-remission therapy is driven by the actual risk of relapse. The German AML Cooperative group demonstrated that MRD persistence on day 16 and the log-difference between MRD positive cells on day 1 and day 16,was an independent prognostic factor affecting CR, EFS, OS and RFS.^53^–^56^ In the same line of research, two different studies^57^,^58^ have established a correlation between the degree of peripheral blood and BM blast clearance as measured on day 14 after induction. In turn, these parameters correlated with achievement of morphological CR at the end of the induction cycle. Levels of MRD, as determined after induction therapy, also seem to correlate with the quality of peripheral recovery at the time of morphologic remission. In a retrospective study including 245 adults with AML, those who achieved CR had detectable MRD less frequently and at lower levels (median, 0.5%; range 0.004% to 3.9%) than patients achieving CR with incomplete platelet or WBC recovery. This finding suggests that failure in the resumption of normal peripheral blood values may result not only from the commonly assumed toxicity to normal progenitors but also from the persistence of residual leukemia. Furthermore, although peripheral blood recovery and MRD level are linked, each of them was an independent prognostic factor impacting on relapse rate, OS and RFS.^3^ MRD status may also serve as a surrogate for optimal biological dosing of chemotherapeutic agents. To explore this hypothesis, we carried out a retrospective analysis of 130 patients who achieved an mCR after one cycle of either standard dose (SDAC) or high doses of cytarabine (HDAC). 59 We observed that the SDAC regimen was associated with a greater MRD-negativity frequency. In 178 patients, who achieved CR after intensive induction, the MRD level assessed at days 16–18 after induction, was associated with outcome. A cutoff of 0.15% was used to identify cases MRD positive. The 5-year RFS was 16% for MRD-positive patients and 43% for patients with no evidence of residual disease (p<0.001).^60^ Thus, a rapid decline in MRD levels after induction therapy may reflect a highly chemo-sensitive disease with a “per se” favorable prognosis.^61^ Early MRD clearance was also prognostic within the intermediate cytogenetic risk group (5-year RFS 15% vs 37%, P= 0.016) as well as for patients with normal karyotype and NPM1 mutations (5-year RFS 13% vs 49%, P=0.02) or FLT3-ITD (3-year RFS rates 9% vs 44%, P=0.016).^60^ The prognostic impact of flow MRD determined post induction^52^,^62^ and post consolidation was subsequently confirmed in several studies. In a large cohort of younger patients, low MRD values distinguished patients with a relatively favorable outcome from those with a high relapse rate, short RFS, and OS. Either in the whole group or in the subgroup with intermediate-risk karyotype, MRD was an independent prognostic factor. Multivariate analysis after cycle 2 confirmed that high MRD values (>0.1% of WBC) were associated with a greater risk of relapse.^63^ These data were confirmed in a large cohort of older patients treated within UK-NCRI protocols. MPFC-MRD negativity, which was achieved in 51% of patients after cycle 1 (C1) (n =286) and 64% of patients after cycle 2 (C2) (n =279), conferred a significantly better 3-year survival from CR (C1: 42% vs26% in MRD-positive patients, P=0.001; C2: 38% vs18%, respectively; P<0*.*001).MPFC-MRD negativity was also associated with a lower relapse rate (C1: 71% vs83% in MRD-positive patients, P= 0.001; C2: 79% vs91%, respectively; P<0*.*001), being the higher risk of early relapse observed in MRD-positive patients (median time to relapse, 8.5 vs17.1 months, respectively).^64^ The authors concluded that post-induction MRD assessment was able to predict disease outcome better than the post-consolidation evaluation. However, also diverging opinions have been published supporting the hypothesis that delayed time-points may be even more informative as compared to earlier ones. Our group has demonstrated^65^ that levels of MRD ≥ 3.5×10^−4^ as measured after consolidation therapy were associated with a high probability of relapse and a short duration of OS and RFS. The prognostic role of MRD positivity after consolidation was confirmed in multivariate analysis. This observation was further challenged in two extended series of 100 and 147 patients^66^,^67^ confirming that the persistence of ≥ 3.5×10^−4^ residual leukemic cells, at the end of consolidation therapy, discriminated between high and low-risk categories. In line with our experience, Kern et al.^55^ reported that the 75^th^ percentile of the MRD log-difference between day 1 and post-consolidation time-point was the sole variable dividing the patients into two groups with significantly different OS. Moreover, Walter et al.^68^ found that MRD assessment at the pre-ASCT time-point correlated with outcome. In 253 consecutive patients receiving myeloablative (MA) ASCT, a three-year estimate of OS were 73% and 32% in MRD negative and MRD positive patients, respectively. The level of residual disease ≥0.1% was considered as MRD positivity. The pre-ASCT time-point and the 0.1% threshold were more recently confirmed in a series of 241patients who received either non-myelo-ablative(NMA) or MA ASCT. Three-year relapse estimates were 28% and 57% for MRD negative and MRD positive NMA patients, and 22% and 63% for MA patients.^69^ The prognostic significance of peri-transplant MRD dynamics was recently confirmed in a series of 279 adults patients who received MA ASCT in first or second remission. Ten-color multiparametric flow cytometry analyses of marrow aspirates were performed before and 28±7 days after transplantation. The 214 MRD negative patients had excellent outcomes, whereas those with MRD positivity before or after ASCT had a high risk of relapse and poor survival.^70^ In order to improve the prediction power of MRD approach, Zhao et al.^71^ exploited a combination of LAIP and WT1. They defined a positive MRD combination as two consecutive positive findings of WT1, MPFC or both, in the same sample, within a year post transplantation. With this dual approach, a higher sensitivity than the single approach was achieved, without loss of specificity. Several studies confirmed a good correlation between MRD detection by MPFC and WT1 analysis, after ASCT.^72^–^73^ In line with this, Rossi et al.^74^ observed comparable results at day +30 post-transplant. However, at day +90 WT1 analysis showed a significantly superior prediction power than MPFC, suggesting that WT1 expression may be more reliable in a long-term MRD follow up.

Selecting an early or delayed time-point might entail the choice of different therapeutic options: the early time-point option may prove useful to identify as soon as possible high risk patients for whom a fast allocation to very intensive treatments is required. For these patients, approaches such as dose dense schedule^75^ and/or ASCT could be incorporated into the upfront treatment strategy.^76^ On the other hand, opponents to this hypothesis raise concerns of potential over-treatment for patients showing a slow blast clearance which can cause MRD to be still positive after induction and negative after consolidation. In our experience^65^,^66^ approximately 30% of patients who are MRD positive after induction, become negative at the end of consolidation; this underlines the impact of a standard consolidation course in rescuing into an MRD negative status a significant proportion of patients. The clinical outcome of these “slow responders” is not significantly different from that of patients who test MRD negative soon after induction. Based on these observations, we hypothesized that the final outcome will rely on the overall debulking effect produced by the whole [induction-consolidation] upfront therapy.^65^,^66^ In our experience, the prognostic significance of post consolidation flow MRD is also maintained in elderly patients. Comparing 149 young and 61 elderly adults we observed that elderly patients reached a post-consolidation MRD negative status less frequently than younger ones (11% *vs* 28%, p=0.009). However, once attained, MRD negativity resulted in a longer 5-year disease-free survival (DFS) both in elderly (57% vs 13%, p=0.0197) and in younger patients (56% vs 31%, p=0.0017). Accordingly, 5 year CIR of both elderly (83% vs 42%, p=0.045) and younger patients (59% vs 24% p=NS) who were MRD positive doubled that of MRD negative ones. Nevertheless, CIR of MRD negative elderly patients was almost twofold higher than that of younger MRD negative ones (42% vs 24%, p=NS).^77^

In the light of the prognostic relevance of MRD detection by MPFC, we tried to optimize risk-assessment of patients with AML by integrating the evaluation of pre-treatment prognosticators and MRD amount at the post-consolidation time-point. 78,79 Of 143 adult patients, those with favorable and intermediate-risk karyotype who were MRD negative had 4-yrs RFS of 70% and 63%, and OS of 84% and 67%, respectively. Patients with favorable and intermediate-risk karyotype who were MRD-positive had 4-yrs RFS of 15% and 17%, and OS of 38% and 23%, respectively (p<0.001 for all comparisons). Likewise, FLT3 wild-type patients achieving a MRD-negative status had a better outcome than those who remained MRD-positive after consolidation (4-yrs RFS 54% vs 17% p<0.0001, OS 60% vs 23% p=0.002). Therefore, patients with favorable risk karyotype, intermediate-risk or FLT3 wild-type had a very different outcome depending on MRD status at the end of consolidation. Doing so, we demonstrated that patients with favorable-risk karyotype or unmutated FLT3, whose course of the disease is conventionally classified as favorable, show a very different outcome depending on MRD status at the end of consolidation.

## Open Issues

### Optimization of molecular MRD monitoring

At the current time, optimized molecular monitoring of AML should be carried out taking into account several technical and practical aspects, such as the patient age and treatment objectives (e.g. disease eradication), best source of sampling (bone marrow or peripheral blood), chosen biomarkers, assay sensitivity (indicated by level of expression of leukemic transcripts relative to the control gene), and kinetics of disease preceding relapse. As to sampling source, Ivey et al. recently demonstrated that the presence of MRD, as determined by quantitation of NPM1^mut^transcripts in peripheral blood, provided significant information on prognostic outcome. BM evaluation, therefore, remains an important adjunct to peripheral blood analysis in patients with AML.^7^

### LAIP reliability

Aberrant phenotypes include LAIPs which some authors claim to be expressed even on normal cells, therefore compromising LAIPs reliability for MRD monitoring. Actually, Rossi et al., in a six-color assay, demonstrated that CD15^+^/CD117^+^ positive cells could also be detected in BM of healthy donors.^80^ In our opinion, the chance to efficiently distinguish leukemic from normal cells increases proportionally with the number of fluorochromes in the assay. In the AML1310 GIMEMA prospective trial, recruiting more than 500 hundred young patients with de novo AML, we detected reliable LAIPs in 91% of the cases, using an 8-color assay (data unpublished).

### Statistical methods for MRD evaluation by MPFC

The statistical methods used for the choice of the best cut-off and time-point is a subject of debate and solutions adopted are quite heterogeneous. Some authors, such as Al Malawi et al.,^60^ used the receiver operating characteristic (ROC) analysis to select cut-offs and time-points. However, this approach requires that time-dependent endpoints (survival estimates) are transformed into binary end points, clinically relevant. Based on this, others prefer to use the maximally selected log-rank test.^52^,^78^,^79^ In our opinion, the latter has some important advantages over ROC analysis. First, there is no need to transform the time-dependent end points. Second, the test calculates an exact cut-off point and provide a P value to substantiate its discrimination power.^81^

### Immunophenotypic shift

Comparison of paired presentation/relapse samples showed instances of selective LAIP changes. These changes consist in reduction/loss or increment/gain of antigens expression in AML. The antigens more frequently lost are CD11b, CD14, CD15, while those more often acquired are CD34 and CD117.^82^–^85^ Our and others’ opinion is that changes between diagnosis and relapse might depend on outgrowth of therapy-resistant sub-clones characterized by immunophenotypic aberrancies distinct from those belonging to the original clone.^86^ The outgrowth of such minor subpopulation(s) until overt relapse, might theoretically be anticipated since diagnosis, if such subpopulations are identified. In this view, appears critical, once again, the number of fluorochromes in the assay. Moreover, these immunophenotypic “shifts” may be correlated with particular molecular and/or cytogenetic “shifts”. Seven patients whose mutational status at diagnosis was determined in cell-sorted sub-fractions, experienced a relapse characterized by changes in the mutation pattern. Actually, the mutations observed at relapse were already present at low frequencies in the primitive CD34^+^CD38^−^populations.^86^ In line with this, Angelini et al.^87^ evaluated a possible correlation between specific LAIPs and the presence of mutations of FLT3 and NPM1. BM samples from 132 newly diagnosed AML patients were analyzed by 9-color MPFC. Within the CD34^+^ population, a small fraction of CD123^+^CD99^+^CD25^+^ cells was identified. The expression of this phenotype in ≥11.7% of the CD34^+^ cells, correlated with the presence of FLT3-ITD mutations, with a specificity and sensibility >90%. CD34^+^CD123^+^CD99^+^CD25^+^ clones were also detectable at presentation in 3 patients who had FLT3wild type/NPM1^mut^ AML and who relapsed with a FLT3mutated/NPM1^mut^AML. In all of the 3 cases, RQ-PCR designed at relapse for each *FLT3*-ITD confirmed the presence of low copy numbers of the mutation in the diagnostic samples.

### Peripheral blood vs BM in MRD monitoring by MPFC

Peripheral blood (PB) is an attractive alternative source for MRD detection, considering that BM collection is a burden for the patients, can be quite traumatic and, in some cases, the aspiration fails (dry tap). Furthermore, PB MRD might have higher specificity due to the relative absence of normal myeloid progenitors in PB. We demonstrated that after induction and consolidation therapy, the findings in BM and PB were significantly concordant.^88^ The cut-off value of residual leukemic cells in PB which correlated with outcome was 1.5×10^−4^. After consolidation, 38 of 50 patients had a level of MRD >1.5×10^−4,^ and 31 (82%) had a relapse. Recently, Zeijlemaker et al.^89^ observed a significant correlation between PB and BM and that MRD detection in PB is more accurate than in BM. With MRD being assessed after induction therapy, the 1-year cumulative incidence of relapse therapy was 29% for PB MRD negative and 89% for PB MRD positive patients (p<0.001). Three-year overall survival was 52% for MRD negative and 15% for positive patients (p=0.034). Similar differences were found after consolidation therapy.

### Leukemic Stem Cell (LSC)

Finally, a lot of attention is being dedicated to the identification of leukemic stem cell (LSC). Targeting LSC represents a very ambitious goal not only for MRD purposes but also for the formidable therapeutic implications. LSC resides within the CD34^+^CD38^−^ cell fraction is responsible for leukemia initiation and relapse because of its self-renewal and repopulating capacity.^90^,^91^ Since LSC is more resistant to chemotherapy than the more mature CD34^+^CD38^+^ progeny, its persistence after chemotherapy may explain treatment failure in MPFC MRD negative AML patients. The expression of LSC-specific markers, such as CD47,^92^ CD123, CD44 and C-type lectin-like molecule 1(CLL-1)^93^,^94^ allows to distinguish LSCs from their normal counterpart. In particular, it was found that CLL-1 expression on CD34^+^CD38^−^is relatively stable between diagnosis and relapse.^93^,^95^ Using the combination CLL-1/CD34/CD38, Van Rhenen et al.^96^ demonstrated that high percentages of residual LCS, as measured at each course of chemotherapy, correlated with shorter patient survival. Moreover, combining LSC and MRD frequencies, 4 patients’ groups, with different survival, were identified. The LSC-/MRD- group had the best prognosis while the LSC+/MRD+ the worst. In order to better quantify LSC both at diagnosis and follow-up, Zeijlemaker et al.^97^ designed a single 8-color detection tube including common markers (CD45, CD34 and CD38), specific markers (CD45RA, CD123, CD33, CD44) and a marker cocktail (CLL-1/TIM-3/CD7/CD11b/CD22/CD56) in one fluorescence channel. The LCS detection tube allows recognizing not only residual cells with an immunophenotype established at diagnosis but also those with emerging immunophenotypes. Additionally, this tube is lower in costs and requires fewer BM materials as compared with a multiple-tubes approach.

## Future Directions

MRD detection may help refine risk-assessment of AML and, therefore, “customize” the therapeutic decision-making process*.* In this view, a comprehensive risk-stratification, generated by integrating the prognostic role of pre-treatment (cytogenetics/genetics) and post-treatment parameters (MRD), might help allocate the majority of patients in a more realistic category of risk. The adjusted risk-allocation might implement selection of a more appropriate post-remission strategy, particularly in regard to ASCT. In conclusion, the current treatment strategy of patients with AML must rely on a rigorous biological characterization at diagnosis to allow high risk patients to be treated intensively and timely submitted to ASCT. For the remainders, estimation of MRD status appears appropriate in order to extrapolate patients at high risk of relapse (MRD positive) for whom ASCT is required to pursue a survival advantage and low risk patients (MRD negative) for whom standard treatments may be adopted, avoiding excessive toxicity that may jeopardize an otherwise favorable clinical outcome.

## Figures and Tables

**Table 1:** Potential molecular targets for MRD

Molecular targets	Frequency
Fusion genes	25–30%
*PML-RARA*	
*CBFB-MYH11*	
*RUNX1-RUNX1T1*	
*MLL-fusion partner*	
Mutations	75%
*NPM1*	
*FLT3*	
*RUNX1*	
*MLL-PTD*	
Overexpression	80%
*WT1*	

*Abbreviation: PML-RARA,* promyelocytic leukemia gene retinoic acid receptor-alpha*; CBFB-MYH11,* core-binding factor subunit beta-myosin heavy chain 11; *RUNX1-RUNX1T1,* runt-related transcription factor 1/runt–related transcription factor 1 translocated to 1; *MLL*, mixed-lineage leukemia; *NPM1*, mutated nucleophosmin1; FLT3, Fms-like tyrosine kinase; *MLL-*PTD, mixed-lineage leukemia partial tandem duplications; WT1, Wilm’s Tumor gene.

**Table 2:** Incidence of LAIPs in AML

Leukemic phenotype	Incidence	Examples
Asynchronous expression	60–70%	CD34 CD14 CD117 CD15
Cross-lineage expression	30–40%	CD19 CD2 CD7
Overexpression	20–30%	CD34 CD13 CD33 CD64 CD15 CD14
Lack of expression	20–30%	DR CD33 CD13
**Overall**	**90–95%**	
